# Transcriptomic and Physiological Analyses Reveal Potential Regulatory Networks of Cadmium Stress Response Mediated by PSR1 in *Chlamydomonas reinhardtii*

**DOI:** 10.3390/cimb48060593

**Published:** 2026-06-04

**Authors:** Yihan Wang, Mengchen Lv, Ying Li

**Affiliations:** 1 Haide College, Ocean University of China, Qingdao 266100, China; wwyh05@163.com; 2 Marine Science Research Institute of Shandong Province, Qingdao 266104, China; charlolle@163.com; 3 State Key Laboratory of Mariculture Biobreeding and Sustainable Goods, Yellow Sea Fisheries Research Institute, Chinese Academy of Fishery Sciences, Qingdao 266071, China; 4 Laboratory for Marine Fisheries Science and Food Production Processes, Qingdao Marine Science and Technology Center, Qingdao 266237, China

**Keywords:** cadmium stress, *Chlamydomonas reinhardtii*, phosphate, *PSR1*, RNA-seq

## Abstract

Cadmium (Cd) is one of the most toxic heavy metals in the environment, and it severely represses photosynthesis, growth, development and nutrient uptake in photosynthetic organisms. Excessive cadmium (Cd) taken up by plants seriously threatens global food security and human health. Therefore, designing an eco-friendly and sustainable strategy that can reduce the accumulation of Cd in plants is a major challenge. Phosphorus (P), as an essential nutrient for plant growth, has been shown to play a pivotal role in mediating Cd-induced stress response. However, the molecular mechanisms underlying the crosstalk between phosphate signaling and Cd stress response remain largely uncharacterized, especially the role of the core phosphate homeostasis regulator Phosphate Starvation Response 1 (PSR1). Here, we used the model green microalga *Chlamydomonas reinhardtii* to investigate the physiological and transcriptomic responses to Cd stress in wild type (WT, CC-125) and *PSR1* loss-of-function mutant (*Crpsr1*, CC-4267). Our results showed that the *Crpsr1* mutant exhibited significantly enhanced Cd tolerance compared with WT under P-sufficient conditions, with a better growth phenotype and a significantly lower Cd accumulation. Transcriptome analysis revealed distinct gene expression profiles between WT and the *Crpsr1* mutant in response to Cd treatment. Gene Ontology (GO) enrichment analysis showed that differentially expressed genes (DEGs) were mainly involved in primary metabolism, protein kinase activity, ion binding and transmembrane transport, which are critical processes for mitigating Cd stress. Notably, key genes associated with iron uptake and homeostasis were significantly upregulated in the *Crpsr1* mutant under Cd stress, indicating a potential regulatory link between PSR1, iron homeostasis and Cd tolerance. Taken together, our findings establish a functional association between the central phosphate signaling regulator PSR1 and Cd stress response in green microalgae, and provide novel candidate genes and regulatory networks for developing engineered microalgae with enhanced Cd phytoremediation capacity.

## 1. Introduction

Cadmium (Cd), a toxic, non-essential heavy metal in ecosystems, is widely distributed by anthropogenic activities, such as phosphorus fertilizer, industrial emissions, mining, and agricultural practices [1,2]. Due to its high solubility, easily mobility, and deadly toxicity, Cd has become a worldwide pollutant. In photosynthetic organisms, Cd is readily absorbed by cells, and excessive Cd accumulation impairs photosynthesis, disrupts nutrient uptake, induces oxidative stress, and ultimately inhibits growth and development [3]. Finally, the non-biodegradable nature of Cd will seriously threaten human health and global food security via the food chain [4,5].

To address Cd pollution, phytoremediation using photosynthetic organisms has emerged as an eco-friendly and cost-effective strategy, compared with high-risk physical and chemical remediation approaches [6]. Recently, microalgae-based bioremediation has emerged as a promising, low-cost and sustainable alternative to conventional wastewater treatment strategies. Unicellular microalgae possess a large surface-to-volume ratio, enabling high-efficiency metal uptake via channel-mediated transport and surface biosorption [7]. Beyond heavy metal removal, microalgae can utilize inorganic nitrogen and P in wastewater for vegetative growth, supporting simultaneous nutrient recovery and pollutant removal. The resulting algal biomass can be further valorized for multiple high-value applications, including biogas production via anaerobic digestion, creating a closed-loop circular system [8]. Notably, unlike vascular plants, microalgae can store excess intracellular P in the form of polyphosphate (PolyP) granules in acidocalcisomes [9]. These PolyP granules not only act as a slow-release P fertilizer for agricultural reuse, but also serve as a key intracellular chelator for heavy metal detoxification, highlighting the tight functional link between P homeostasis and heavy metal tolerance in microalgae [10,11]. This dual function of algal PolyP provides a novel molecular target for engineering algal strains with enhanced synchronous wastewater remediation and nutrient recovery capacity.

The unicellular green alga *Chlamydomonas reinhardtii* is an ideal model organism for investigating the crosstalk between P homeostasis and heavy metal stress response, owing to its rapid life cycle, well-annotated reference genome, and widespread use in cellular physiology and molecular biology studies [12,13]. Previous studies have characterized the toxic effects of Cd on Chlamydomonas strains: Cd exposure induces significant growth inhibition [14,15], ultrastructural cellular alterations including cytoplasmic vacuolization, starch accumulation and cytoplasmic electron-dense granules [16,17], abnormal development of membranous organelles, increased relative cell size [14,17], and severe inhibition of photosynthetic activity [18,19,20,21,22]. However, the upstream transcriptional regulatory networks governing Cd tolerance and detoxification in *C. reinhardtii* remain largely uncharacterized, especially the regulatory role of master nutrient homeostasis regulators in this process.

Phosphorus (P) is an essential macronutrient for plant growth and development, and also plays vital roles in numerous types of stress resistance [23,24]. The interaction processes between Phosphorus homeostasis and stress response are tightly interconnected, and significantly affect plant growth and survival [25]. Excessive Cd in soils adversely represses normal plant growth. Exogenous phosphorus application may be a potential strategy to alleviate Cd stress in plants. In soybean, phosphorus treatment can induce the expression of genes involved in stress response, root development, and metal transport, thereby reducing Cd uptake and enhancing photosynthesis [26,27]. Interestingly, the application of exogenous phosphorus can suppress the expression of *OsHMA2*, *OsIRT1*, and *OsABCC1*, which are genes for Cd transporters, suggesting that phosphorus enhances Cd resistance in rice by reducing Cd accumulation through the regulation of metal transporter genes [28]. In summary, these results suggest that optimizing phosphorus application can mitigate the adverse impacts of Cd toxicity, thereby promoting plant growth and stress resistance.

Cd stress can modulate plant Pi signaling pathways by activating numerous PSR genes such as *OsIPS1*, *OsPT6*, and *OsPT10* in rice [29]. In this response process, the transcription factors PHOSPHATE STARVATION RESPONSE proteins (PHRs), a class of MYB-CC family genes, are identified as the central regulators of Pi-starvation signaling and P homeostasis in land plants and green algae [30,31]. Moreover, *PHRs* have been widely found to play important roles in interactions between Pi signaling and other abiotic stress responses in plants [32,33,34,35]. Here, the green microalga is the ideal experimental plant for Cd stress due to its rapid growth rates and Cd binding site on the cell surface [36]. Previous studies have explored the expression of genes involved in oxidative, cell wall-related, and photosynthesis-related phosphorus and metal iron homeostasis. These are dynamic altered after Cd treatment in the green microalga *Chlamydomonas* (*Chlamydomonas reinhardtii*). *Phosphate Starvation Response 1* (*PSR1*), the homeolog gene of *OsPHR2* and *AtPHR1,* may participate in Cd response networks, as the *psr1* mutant shows less Cd accumulation and resistance to Cd treatment compared with the WT [37,38,39]. However, the molecular mechanism by which PSR1 mediates Cd stress tolerance remains unclear.

In order to better understand the mechanism of *CrPSR1*-regulated Cd tolerance, we explored the mechanisms at the transcriptomic and physiological levels. The results indicate that the *Crpsr1* mutant has higher Cd tolerance than the WT under P-sufficient conditions, and the transcriptome analysis revealed remarkable changes in gene expression profiles related to metabolism, protein kinase activity, ion binding and transmembrane transport between the WT and *Crpsr1* mutant in response to Cd treatment. Taken together, our findings identify the key players involved in the Cd detoxification processes and provide new insights into the regulatory mechanisms of central phosphate signaling-mediated Cd response in microalgae. This study provides a novel regulatory link between phosphate signaling, iron homeostasis, and Cd tolerance in green algae, which has been completely unexplored in previous work. Our findings also offer a new genetic target (PSR1) for engineering microalgae with enhanced Cd phytoremediation capacity and stable phosphorus recovery from wastewater, which has important theoretical and practical significance.

## 2. Materials and Methods

### 2.1. Strains, Growth Conditions and Cd Treatment

The *Chlamydomonas reinhardtii* wild-type strain CC-125 (WT) and *psr1 loss-of-function mutant strain CC-4267 (Crpsr1)* were obtained from the Chlamydomonas Resource Center [40]. Cells were cultured in a standard Tris-acetate-phosphate (TAP) medium at pH 7.0 under continuous illumination (50 μmol photons/m^2^/s) on a rotating platform (150 rpm) at 24 °C. TAP medium contained: 2.42 g/L Tris, 0.375 g/L NH_4_Cl, 0.1 g/L MgSO_4_·7H_2_O, 0.05 g/L CaCl_2_·2H_2_O, 10.5 mg/L K_2_HPO_4_, 5.4 mg/L KH_2_PO_4_, 1 mL/L glacial acetic acid, and 1 mL/L Hunter’s Trace Stock Solution [41]. For cadmium treatment experiments, cells were cultured in modified TAP medium where inorganic phosphate was replaced by β-glycerophosphate (TAgP) to avoid Cd-P complex formation, and treated with 50 μmol/L CdCl_2_ for 3 days, and 0 μmol/L Cd treatment as control.

### 2.2. Measurement of Total Phosphorus

Total phosphorus content was determined using the molybdenum-antimony anti-spectrophotometric method [42]. First, 10 mL of algal culture was harvested by centrifugation (3000× *g*, 5 min), washed twice with deionized water, and dried at 80 °C to constant weight. The dried algal powder was digested with 5 mL of H_2_SO_4_-H_2_O_2_ at 300 °C until the digest was clear and transparent. After cooling, the digest was diluted to 50 mL with deionized water. Then, 1 mL of the diluted digest was mixed with 4 mL of deionized water and 1 mL of molybdenum-antimony anti-color reagent, and incubated at 30 °C for 30 min. The absorbance was measured at 700 nm using a UV-Vis spectrophotometer. A standard curve was prepared using KH_2_PO_4_ standard solution to calculate the total phosphorus content of the samples. Three technical replicates were performed for each biological sample.

### 2.3. Measurement of Cell Density and Chlorophyll Content

The cell density was estimated by OD_750_ in a spectrophotometer. For chlorophyll a+b content, 1000 μL of culture was collected into a 1.5 mL centrifuge tube and centrifuged at 21,000× *g* for 5 min. The precipitate was dissolved in 1000 μL of 100% methanol for 10 min in darkness. After centrifugation at 21,000× *g* for 5 min, 200 μL of supernatant was used to measure absorbance values on a 96-well plate by using a fluorimeter at 665 and 652 nm.

### 2.4. Measurement of Cd Content

The Cd content of the WT and the *Crpsr1* mutant was measured via inductively coupled plasma mass spectrometry (Agilent 7700 series, Santa Clara, CA, USA) as described [22]. The sample pretreatment method was the same as the measurement of total phosphorus.

### 2.5. RNA Sequencing and Data Analysis

Total RNA was extracted using a Rneasy plant mini kit (Qiagen, Hilden, Germany), and three independent biological replicates were used for each line. Library construction for RNA and sequencing was carried out using the Illumina NovaSeq X plus platform with paired-end (2 × 150 bp) sequencing.

Transcriptome data were prepared as described in a previous study [42,43]. Briefly, paired-end reads for each individual were mapped against the *Chlamydomonas reinhardtii* reference genome (*Chlamydomonas reinhardtii CC-4532 v6.1*) using HISAT2 (version 2.1.0). TPM (transcripts per million) values were calculated by String Tie (version 1.3.4b) with default parameters. Differential expression analysis was carried out by DESeq2 (version 1.32.0). Differential expression with *p* value < 0.05 was considered a significant threshold for detecting differentially expressed genes (DEGs), log_2_(fold change) ≥ 2 and log_2_(fold change) ≤ −0.5 were considered to indicate up-regulation and down-regulation, respectively. Gene ontology (GO) analysis was performed using agriGO v2.0. Significantly enriched GO items were filtered by *p* value < 0.05.

### 2.6. Quantitative RT-PCR

Reverse transcription was performed using 2 μg of total RNA and M-MuLV Reverse Transcriptase (NEB) according to the manufacturer’s instructions. Quantitative PCR (qPCR) was performed using the Vazyme Taq Pro Universal SYBR qPCR Master Mix on the LightCycler480 machine (Roche Diagnostics, Basel, Switzerland) according to the manufacturer’s instructions. Three biological replicates were performed for each gene. The *Actin* gene was used as an internal control. The primers used are listed in Supplementary Table S1.

### 2.7. Statistical Analysis

Differences between groups were analyzed using two-way analysis of variance (ANOVA) in GraphPad Prism 8.0. Differences were considered significant when *p* was less than 0.05. Different letters indicate significant differences determined by Tukey’s test.

## 3. Results

### 3.1. Phenotypic and Physiological Changes Between WT and Crpsr1 Mutant upon Cd Stress

**Figure 1:**
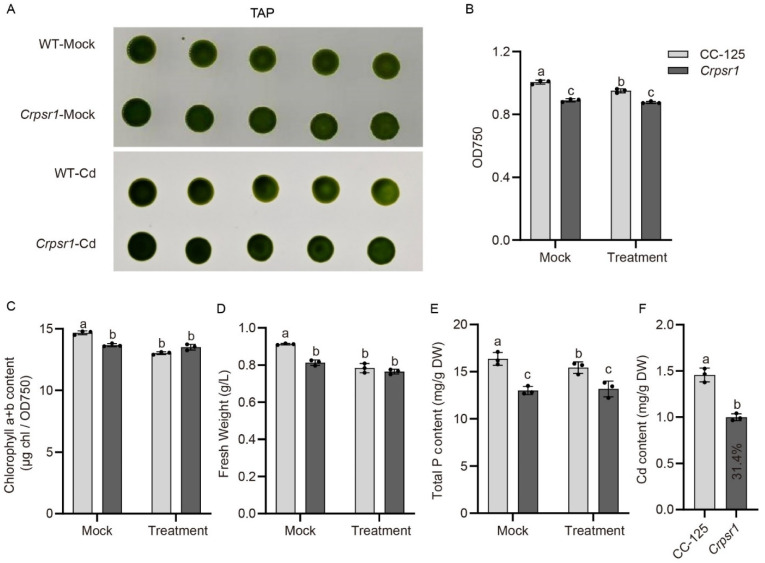
**Phenotypic and physiological changes between WT and the *Crpsr1* mutant under Cd stress conditions.** (**A**) Growth of CC-125 (WT) and the *Crpsr1* mutant strain in the TAP (with Pi supply) media. Colonies from left to right depict a series of dilutions. The panel at the right shows the growth curves of WT and the *Crpsr1* mutant under Pi-supply conditions. (**B**) Biomass estimation after 3 days of Cd treatment. Error bars represent ± SD (*n* = 3). (**C**) Chlorophyll a+b content of WT and the *Crpsr1* mutant after 3 days of Cd treatment. Error bars represent ± SD (*n* = 3). (**D**) Fresh weight of WT and the *Crpsr1* mutant after 3 days of Cd treatment. Error bars represent ± SD (*n* = 3). (**E**) Total P content of WT and the *Crpsr1* mutant after 3 days of Cd treatment. DW, Dry Weight. Error bars represent ± SD (*n* = 3). (**F**) Cd content of the WT and the *Crpsr1* mutant after 3 days of Cd treatment. DW, Dry Weight. Error bars represent ± SD (*n* = 3). Samples were collected from three independent experiments. Different letters in (**B**–**F**) show a significant difference (*p* < 0.05) according to Tukey’s test. Different lowercase letters (a, b, c) indicate statistically significant differences between groups (*p* < 0.05, one-way ANOVA followed by Tukey’s post-hoc test). Bars with the same letter are not significantly different from each other.

To clarify the regulatory role of PSR1 in Cd stress response, we first characterized the phenotypic and physiological changes in wild type (WT, CC-125) and *Crpsr1* mutant under 50 μmol/L Cd treatment for 3 days in P-sufficient TAgP medium (Figure 1). Under mock (Cd-free) conditions, the *Crpsr1* mutant showed an obvious growth defect compared with WT, which is consistent with the core function of PSR1 in maintaining phosphate homeostasis and vegetative growth of *Chlamydomonas reinhardtii*. After 3 days of Cd stress treatment, the WT exhibited severe growth inhibition compared with mock conditions, but the *Crpsr1* mutant displayed no significant difference in Cd stress than under control conditions (Figure 1A). Following the Cd exposure, the *Crpsr1* mutant exhibited no reduction in biomass (OD_750_), chlorophyll a+b content, fresh weight, or total phosphorus content compared to normal conditions, whereas these key indicators were obviously reduced under Cd stress in the WT (Figure 1B–E, *p* < 0.05). Then, we measured the Cd content in the WT and the *Crpsr1* mutant, and the results showed that the Cd content in the *Crpsr1* mutant was significantly reduced by about 31.4% compared to the WT (Figure 1F, *p* < 0.001). Taken together, these results collectively demonstrated that loss-of-function of *PSR1* significantly enhanced the tolerance of *C. reinhardtii* to Cd stress under P-sufficient conditions.

### 3.2. Quality Assessment of Transcriptome Sequencing and Global Gene Expression Profiling

**Figure 2:**
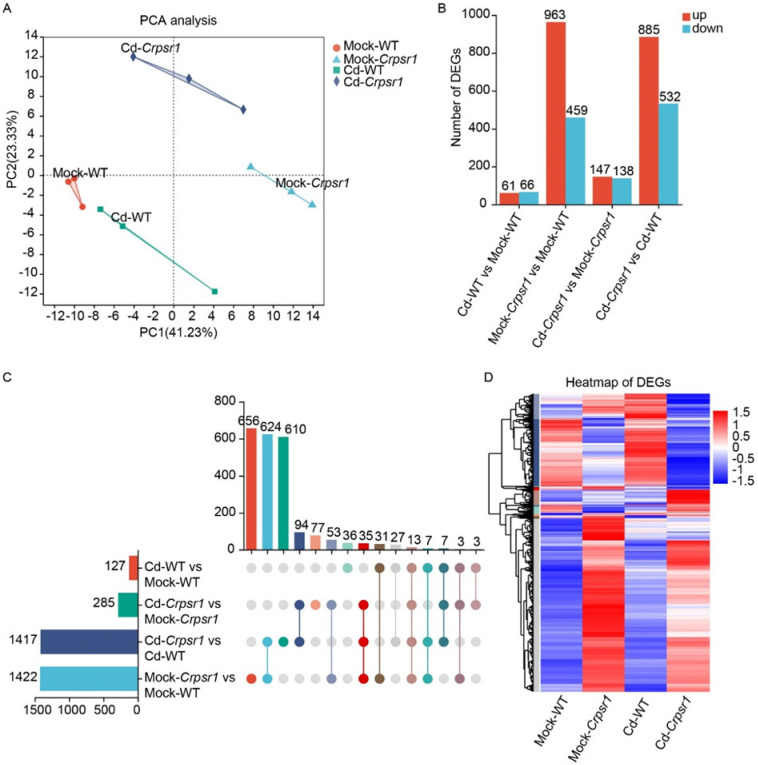
**Transcriptome analysis of WT (CC-125) and the *Crpsr1* mutant under Cd stress.** (**A**) PCA plot indicating the overall distribution and separation of each group. (**B**) The number of upregulated and downregulated DEGs in WT and the *Crpsr1* mutant after 3 days of Cd treatment. The total numbers of upregulated and downregulated genes at each group are shown in bar diagrams. (**C**) UpSet plots of the number of upregulated and downregulated DEGs were identified among the different groups demonstrating different expression patterns. (**D**) Heatmap analysis of all DEGs.

Based on the physiological differences observed between the WT and *Crpsr1* mutant, further investigation is warranted to explore the molecular mechanisms underlying *CrPSR1*-mediated Cd response. Three biological replicate samples of WT (CC-125) and *Crpsr1* mutant after 50 μM Cd treatment for 3 days under Pi-sufficient growth conditions and corresponding controls (without Cd treatment) were subjected to a transcriptomic analysis using RNA-Seq. A total of 12 libraries were sequenced using the NovaSeq X Plus platform (PE150) using NovaSeq Reagent Kit. In summary, after removing the low-quality sequences (quality scores < 25), 79.22 Gb of clean data was obtained, representing approximately 5.98 Gb clean data per sample. All the RNA-seq data showed high quality, with the average Q30 value being higher than 94.33%. The mapping rates for the cleaned sample reads aligned against the reference genome (*Chlamydomonas reinhardtii CC-4532 v6.1*), and the mapping rate ranged from 97.11 to 97.58% for all samples. The principal component analysis (PCA) of gene expression profiles across each condition revealed a distinct segregation of the samples, and PC1 and PC2 explained 41.23% and 23.33% of the gene expression variance, respectively (Figure 2A).

### 3.3. Identification and Functional Analysis of CrPSR1-Regulated Genes Under Cd Stress

To identify differentially expressed genes (DEGs) between the WT (CC-125) and the *Crpsr1* mutant after Cd treatment, the expression level of each transcript was calculated and quantified according to the TPM values (transcripts per million mapped reads) by using the DESeq2. A *p* value < 0.05 was considered a significant threshold for detecting DEGs, and log2(fold change) ≥ 2 and log2(fold change) ≤ 0.5 indicated up-regulation and down-regulation, respectively. Subsequently, our analysis identified 3251 DEGs in all 4 groups (127 DEGs in Group 1: Cd-WT vs. Mock-WT, 1422 DEGs in Group 2: Mock-*psr1* vs. Mock-WT, 285 DEGs in Group 3: Cd-*psr1* vs. Mock-*psr1*, and 1417 DEGs in Group 4: Cd-*psr1* vs. Cd-WT), of which 2056 DEGs were upregulated and 1195 DEGs were downregulated, respectively (Figure 2B).

**Figure 3:**
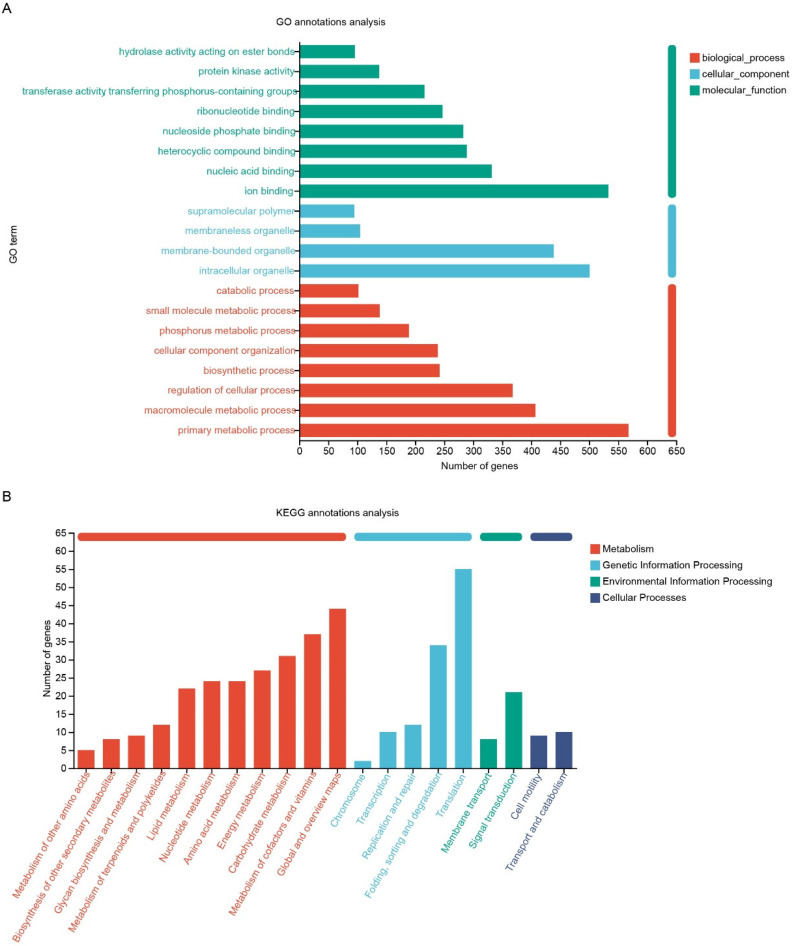
**GO and KEGG analysis illustrating the differential expression of genes.** (**A**) Gene ontology (GO) analysis of the biological process of DEGs detected in WT and the *Crpsr1* mutant after 3 days of Cd treatment. (**B**) KEGG analysis of the biological process of DEGs detected in WT and the *Crpsr1* mutant after 3 days of Cd treatment.

Interestingly, upset plots of the number of upregulated and downregulated DEGs are identified among the different groups demonstrating different expression patterns (Figure 2C). Heatmap analysis of all DEGs also showed that there were significant changes after the Cd treatment in the *Crpsr1* mutant compared with normal conditions, although there were fewer DEGs in the WT under Cd stress (Figure 2D). In order to reveal the functional changes between the WT and the *Crpsr1* mutant under Cd stress, we performed functional enrichment analysis, including GO and KEGG enrichment analysis (Bonferroni-corrected, *p* < 0.05), by using Goatools (v1.3.1) and Python SciPy software (v1.11.4), respectively (Figure 3). The results showed that most DEGs were significantly enriched in the GO terms of primary metabolic process (GO:0044238), ion binding (GO:0043167), intracellular organelle (GO:0043229), membrane-bounded organelle (GO:0043227), macromolecule metabolic process (GO:0043170), regulation of cellular process (GO:0050794), biosynthetic process (GO:0009058), and phosphorus metabolic process (GO:0006793) (Figure 3A). In addition, the KEGG analysis also suggested that the pathways related to Lipid metabolism, Nucleotide metabolism, Amino acid metabolism, Energy metabolism, Carbohydrate metabolism, Metabolism of cofactors and vitamins, Transcription, Translation, Replication and repair, Membrane transport, and Signal transduction were significantly enriched (Figure 3B).

As described before, a combination of alleles of *OsNRAMP5^LAA^* and *OsHMA3^LAA^* can reduce Cd accumulation [22]. We searched the *Chlamydomonas reinhardtii* reference genome and identified the homolog genes of the *OsNRAMP5* and *OsHMA3* as *Cre05.g248300* (*CrNRAMP4*) and *Cre10.g424775* (*CrCTP2*), respectively. The expression levels of these two genes were significantly induced (*p* < 0.05) in the *Crpsr1* than the WT under Cd stress, suggesting that the Cd uptake and homeostasis were different in the *Crpsr1* mutant (Supplementary Table S2). Taken together, these results indicated that the *CrPSR1* might extensively participate in the Cd stress response processes.

**Figure 4:**
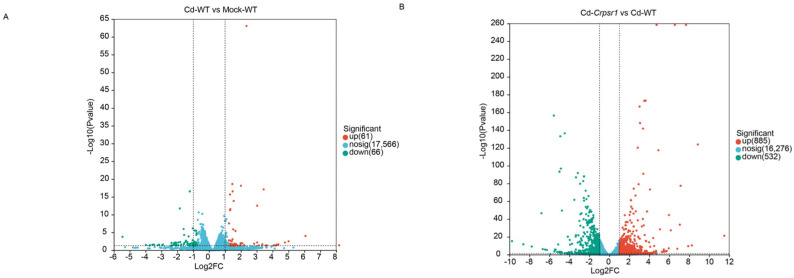
**Volcano plot illustrating the differential expression of genes.** (**A**) Volcano plot illustrating the differential expression of genes in the WT after Cd treatment. (**B**) Volcano plot illustrating the differential expression of genes in the WT compared with the *Crpsr1* mutant after Cd treatment.

**Figure 5:**
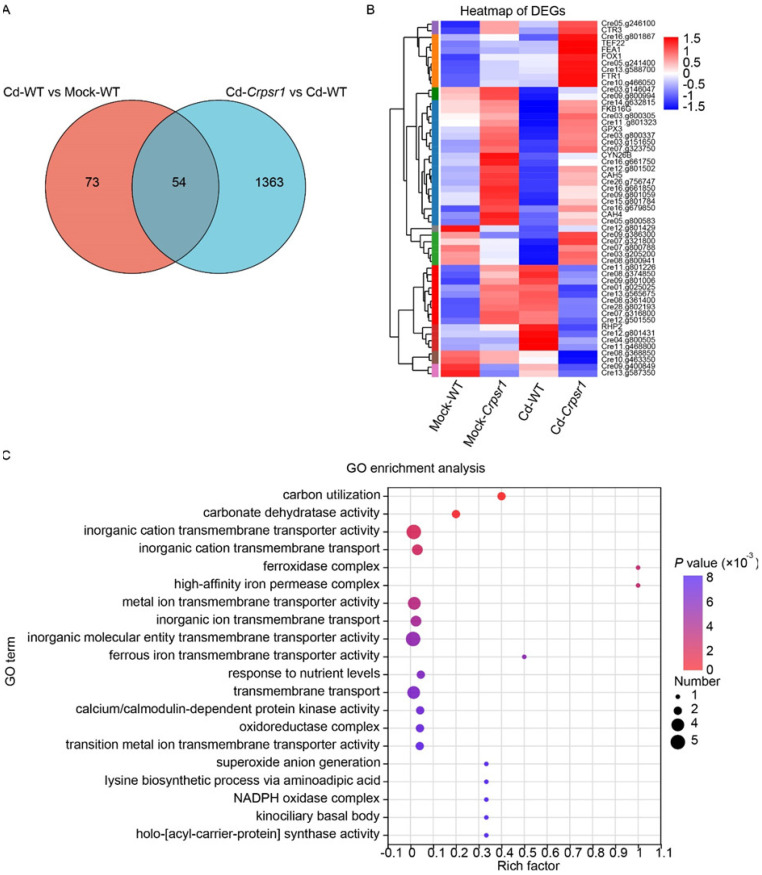
***CrPSR1* negatively regulate Cd resistance by inducing iron homeostasis genes.** (**A**) Venn diagram representing DEGs regulated by Cd (top, left), *Crpsr1* (top, right) and *CrPSR1*-mediated Cd response genes. (**B**) Heatmap analysis of 54 DEGs identified from coregulated by *CrPSR1* and Cd stress. (**C**) GO enrichment analysis shows top 20 terms of 54 DEGs.

In order to further explore the regulatory network controlled by *Crpsr1* under Cd stress, we categorized the DEGs in the WT under Cd treatment as Cd stress response genes, and between the WT and the *Crpsr1* mutant under Cd conditions as *CrPSR1*-regulated genes. We identified 127 (61 upregulated and 66 downregulated genes) and 1417 (885 upregulated and 532 downregulated genes) DEGs between WT and the *Crpsr1* mutant under normal and Cd conditions, respectively (Figure 4A,B). Then, we constructed a Venn diagram to identify the DEGs regulated by *CrPSR1* under Cd stress. As a result, 54 DEGs were found as potential targets (Figure 5A). Heatmap analysis showed that several DEGs belonging to iron uptake and metabolism gene families were activated after Cd exposure in the WT, such as Fe-assimilation protein FEA1, Multicopper ferroxidase FOX1 and Iron transporter FTR1. However, the expression levels of these genes were significantly increased in the *Crpsr1* mutant compared with WT under Cd stress (Figure 5B). Go analysis showed that most DEGs are enriched in the processes of inorganic cation transmembrane transporter activity (GO:0022890), metal ion transmembrane transporter activity (GO:0046873), calcium/calmodulin-dependent protein kinase activity (GO:0004683), and oxidoreductase complex (GO:1990204) (Figure 5C and Supplementary Table S3). Notably, a large set of genes known to be involved in iron accumulation and signaling were strongly induced in the *Crpsr1* mutant respect to the WT under Cd treatment, suggesting that *CrPSR1* negatively regulates the induction of the metal uptake pathways and further rescues the tolerance to Cd exposure.

### 3.4. RT-qPCR Validation of Transcriptome Sequencing Results

**Figure 6:**
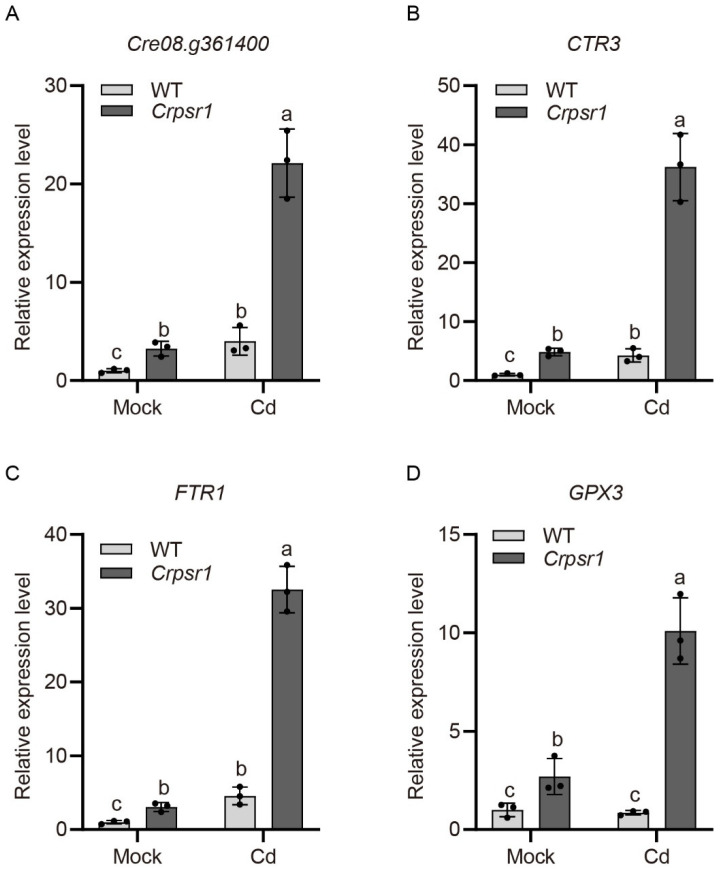
**RT-qPCR analysis to validate the transcriptome sequencing results.** (**A**–**D**) Relative expression levels of 4 randomly selected genes. Three biological replicates were performed for each gene. Error bars represent ± SD (*n* = 3). Samples were collected from three independent experiments. Different letters in (**A**–**D**) show a significant difference (*p* < 0.05) by Tukey’s test. Different lowercase letters (a, b, c) indicate statistically significant differences between groups (*p* < 0.05). Bars with the same letter are not significantly different from each other.

To verify the reliability of the RNA-seq data, we randomly selected four DEGs (*Cre08.g361400*, *CTR3*, *FTR1*, *GPX3*) for RT-qPCR validation (Figure 6). The results showed that the expression trends of these four genes detected by RT-qPCR were completely consistent with the transcriptome sequencing results. Under Cd stress, the expression levels of *Cre08.g361400*, *CTR3*, *FTR1* and *GPX3* were significantly higher in the *Crpsr1* mutant than in WT (*p* < 0.05), which matched the upregulation trend identified by RNA--seq. The high consistency between RT-qPCR and RNA-seq results confirmed the accuracy and reliability of our transcriptome data. The primers used were detailed in Supplementary Table S1.

## 4. Discussion

### 4.1. Loss of PSR1 Function Confers Enhanced Cd Tolerance in C. reinhardtii Under P-Sufficient Conditions

In this study, we systematically characterized the phenotypic and physiological responses of the WT and *Crpsr1* mutants to Cd stress, and found that the mutation of PSR1 significantly enhanced Cd tolerance in *C. reinhardtii* under P-sufficient conditions. Our results revealed a notable growth phenotype of the *Crpsr1* mutant that was dependent on environmental conditions. Under normal (Cd-free) growth conditions, the *Crpsr1* mutant exhibited significant growth retardation, lower biomass, chlorophyll content, total P accumulation, and Cd content compared with the WT, which is consistent with the canonical function of PSR1 as a core regulator of P uptake and homeostasis in *C. reinhardtii*. However, under Cd exposure, all these physiological indicators in WT were severely reduced, while the *Crpsr1* mutant maintained stable growth, photosynthetic pigment content and P homeostasis, with no significant difference from the mock control. This might be related to the fact that cadmium can disrupt the already strong phosphorus absorption system present in the WT. Cd has been widely reported to directly impair the expression and activity of high-affinity phosphate transporters in photosynthetic organisms, thereby disrupting the active phosphate uptake system that is highly functional in WT cells, leading to a significant reduction in intracellular P accumulation under Cd stress [37,39]. In contrast, the *Crpsr1* mutant exhibits constitutively low phosphate uptake activity due to the loss of *PSR1* function, making its intracellular P homeostasis less sensitive to Cd-induced damage and thus maintaining stable P content under Cd exposure [44]. The loss of *PSR1* function can lead to a constitutive pre-activated stress state in algal cells. Previous studies have confirmed that the *psr1* mutant constitutively expresses partial phosphate starvation response and abiotic stress response genes even under P-sufficient conditions, which is known as stress priming [30,44]. This pre-activated stress response network primes the cells to cope with subsequent Cd toxicity, enabling the *Crpsr1* mutant to initiate a more rapid and robust detoxification response upon Cd exposure, compared with the weak and passive stress response in WT [45].

### 4.2. PSR1 Modulates Cd Stress Response via Remodeling Transcriptional Programs Linked to Iron

To decipher the molecular mechanism underlying the enhanced Cd tolerance of the *Crpsr1* mutant, we performed comparative transcriptomic analysis between the WT and *Crpsr1* mutant under Cd stress. We found that the WT had only 127 differentially expressed genes in response to Cd treatment, whereas the *psr1* mutant underwent extensive and robust transcriptional reprogramming under the same stress conditions, with a total of 1417 differentially expressed genes identified between the Cd-treated *psr1* mutant and Cd-treated wild type. This difference indicates that the WT has a very weak and passive response to Cd stress under our experimental conditions, while the loss of *PSR1* function unlocks an active and robust transcriptional response to Cd exposure, which is the core molecular basis for the enhanced Cd tolerance of the *Crpsr1* mutant. Notably, our analysis identified that the core genes of the high-affinity iron uptake system, including *FEA1*, *FOX1*, and *FTR1*, were significantly and robustly upregulated in the *Crpsr1* mutant under Cd stress, while only slight induction was observed in the WT (Figure 5B). This finding reveals a negative regulatory role of *PSR1* in iron homeostasis in *C. reinhardtii*, and provides a mechanistic explanation for the enhanced Cd tolerance of the *Crpsr1* mutant. Cd and iron exhibit similar physicochemical properties and compete for the same transporters in plants [22,45]. Cd exposure has been widely reported to trigger a physiological state in photosynthetic organisms which is defined as pseudo-iron deficiency. This state arises as Cd occupies the iron-binding sites on transmembrane transporters that include the IRT and FTR families, which inhibits cellular iron uptake and disrupts iron-dependent metabolic processes that include chlorophyll biosynthesis and photosynthetic electron transport. These processes are widely recognized as the primary drivers of Cd-induced growth inhibition in photosynthetic species [39,46]. In rice species that are a major staple crop worldwide, enhanced iron uptake that is achieved via overexpression of iron transporters has been found to significantly reduce Cd accumulation in plant tissues and alleviate Cd-induced toxicity. These findings confirm that activation of the high-affinity iron uptake system is an effective and conserved strategy to mitigate Cd stress in photosynthetic organisms [22,47,48]. In *C. reinhardtii*, the FEA1-FOX1-FTR1 cascade constitutes the core high-affinity iron uptake system [46]. Our results showed that this cascade was markedly activated in the psr1 mutant under Cd stress. We speculate that this activation may enhance Cd tolerance through two potential mechanisms: (1) increasing iron competition for transporter binding sites, thereby reducing Cd influx; (2) alleviating Cd-induced pseudo-iron deficiency, which helps maintain chlorophyll stability. This is consistent with our physiological observation that chlorophyll content in the psr1 mutant was less affected by Cd stress. GO enrichment analysis further supported this link, showing significant enrichment of metal ion transmembrane transporter activity in the DEGs.

Beyond iron homeostasis, homologs of key Cd transporters (CrNRAMP4 and CrCTP2) were also differentially expressed in the *CrPSR1* mutant under Cd stress. CrNRAMP4 is homologous to OsNRAMP5, the major Cd uptake transporter in rice [49,50], while CrCTP2 is homologous to OsHMA3, which mediates vacuolar Cd sequestration [51]. This suggests that *Psr1* may also directly regulate Cd uptake and detoxification processes, acting synergistically with iron homeostasis modulation to enhance Cd tolerance [39,52]. Functional enrichment analysis further showed that DEGs were significantly enriched in primary metabolism, protein kinase activity and stress signal transduction pathways (Figure 3). Cd stress often disrupts core metabolic processes in microalgae, and metabolic reprogramming is critical for stress adaptation [37,38]. The enrichment of these pathways in the psr1 mutant indicates that loss of *Psr1* function helps maintain metabolic stability under Cd stress. Additionally, calcium/calmodulin-dependent protein kinase (CCaMK), a key regulator of abiotic stress responses [53,54], was also enriched, suggesting its potential involvement in *Psr1*-mediated Cd tolerance. Taken together, these results demonstrate that *Psr1* regulates Cd stress response through a multilayered transcriptional network, coordinately modulating metal transport, nutrient homeostasis, metabolism and stress signaling.

### 4.3. Combining Pi Signaling and Iron Uptake to Develop Engineering Microalgae for Cd Tolerance

Our findings provide a novel genetic target and robust theoretical basis for the rational engineering of microalgal strains tailored for efficient wastewater bioremediation. Industrial and municipal wastewater frequently harbors high concentrations of toxic cadmium (Cd) and excessive phosphorus (P), two major pollutants that drive widespread aquatic ecosystem damage, water quality deterioration, and eutrophication in global water bodies [55,56]. Conventional physicochemical wastewater treatment technologies are often unable to achieve synchronous heavy metal removal and bioavailable P resource recovery, and most of these methods also suffer from high operational costs, complex processing steps, and the risk of secondary pollution [57,58]. In contrast, microalgae-based bioremediation has emerged as a sustainable, low-cost, and circular alternative, as microalgae can simultaneously assimilate inorganic nutrients for growth and sequester heavy metals from aqueous environments through multiple detoxification pathways [36,59]. Previous studies have successfully engineered *Chlamydomonas reinhardtii* strains with enhanced P accumulation capacity via constitutive overexpression of PSR1, the master transcriptional regulator of phosphate homeostasis, and these engineered strains have been validated to achieve efficient P recovery from real wastewater streams [42,60]. Our current study demonstrates that loss-of-function mutation of PSR1 can simultaneously enhance Cd tolerance and maintain stable intracellular P homeostasis in *C. reinhardtii*, which provides a new and facile genetic engineering strategy for constructing microalgal strains that can achieve synchronous Cd removal and P recovery from complex industrial and municipal wastewater.

The Pi-starvation response genes are regulated by the *Crpsr1*, including PTA/PTB transporters and PHO1/PHOX proteins, and the changes in the expression levels of *PTB8*, *PTB12*, *PHO1* and *PHOX* genes under Cd stress in WT were not significant (Supplementary Table S2). However, the expression level of *PTB12*, a high affinity phosphate transporter, was upregulated by 2.73-fold in the *Crpsr1* mutant compared with WT under Cd stress, indicating that phosphate uptake might be involved in the Cd stress tolerance.

By increasing *PSR1* expression in the algae under *Crptc1* conditions, engineered strains called SPAO (super polyP accumulating organism) are produced. By combining the approaches of enhancing Pi-starvation signaling and accumulating Pi in vacuoles in *Chlamydomonas reinhardtii*, the SPAOs could enhance the P-removal from wastewater [42]. Interestingly, our results demonstrate that *Crpsr1* serves as a key regulator connecting Pi signaling and Cd tolerance in *Chlamydomonas reinhardtii*. Similarly to the developed SPAO approach, we intend to create engineered strains that could resist to Cd exposure. Based on this regulatory module, we propose that combinatorial engineering of Pi homeostasis and iron transport pathways via overexpressing the core iron transporters under a *Crpsr1* mutant background will generate microalgal strains with robust Cd tolerance and low Cd accumulation.

## 5. Conclusions

This study integrated transcriptomic and physiological analyses to investigate the regulatory function of *CrPSR1* under Cd stress in *Chlamydomonas reinhardtii*. We demonstrated that loss-of-function of *CrPSR1* confers significantly enhanced Cd tolerance in *C. reinhardtii* under P-sufficient conditions, a phenotype that is supported by the maintenance of stable growth, photosynthetic pigment content, intracellular phosphorus homeostasis, and less Cd accumulation in the mutant under Cd exposure. Transcriptomic profiling demonstrated that *CrPSR1* extensively remodels gene expression in response to Cd exposure, with significant alterations in genes associated with metal ion transport, protein kinase activity, ion binding, and primary metabolic processes. Mutation of *CrPSR1* strongly activates the expression of iron-homeostasis genes, indicating that *CrPSR1* acts as a negative regulator of the iron uptake pathway, and its mutation alleviates Cd toxicity via the activation of iron homeostasis. Our results established a direct functional link between Pi signaling and Cd tolerance, highlighting *CrPSR1* as a central hub coordinating nutrient balance and heavy metal resistance in green algae.

Taken together, our work provides novel molecular insights into the regulatory mechanisms underlying Cd tolerance in microalgae, and offers promising genetic targets for engineering microalgae with enhanced heavy metal recovery and phytoremediation capacity, thereby supporting applications in environmental remediation and sustainable agriculture.

## Data Availability

All sequencing data sets are available in the NCBI BioProject database under accession number PRJNA1444483.

## Supplementary Materials

The following supporting information can be downloaded at: https://www.mdpi.com/article/10.3390/cimb48060593/s1.
